# Adaptive Significance of the Formation of Multi-Species Fish Spawning Aggregations near Submerged Capes

**DOI:** 10.1371/journal.pone.0022067

**Published:** 2011-07-08

**Authors:** Mandy Karnauskas, Laurent M. Chérubin, Claire B. Paris

**Affiliations:** 1 Division of Marine Biology and Fisheries, Rosenstiel School of Marine and Atmospheric Science, University of Miami, Miami, Florida, United States of America; 2 Division of Meteorology and Physical Oceanography, Rosenstiel School of Marine and Atmospheric Science, University of Miami, Miami, Florida, United States of America; 3 Division of Applied Marine Physics, Rosenstiel School of Marine and Atmospheric Science, University of Miami, Miami, Florida, United States of America; University of Hull, United Kingdom

## Abstract

**Background:**

Many fishes are known to spawn at distinct geomorphological features such as submerged capes or “promontories,” and the widespread use of these sites for spawning must imply some evolutionary advantage. Spawning at these capes is thought to result in rapid offshore transport of eggs, thereby reducing predation levels and facilitating dispersal to areas of suitable habitat.

**Methodology/Principal Findings:**

To test this “off-reef transport” hypothesis, we use a hydrodynamic model and explore the effects of topography on currents at submerged capes where spawning occurs and at similar capes where spawning does not occur, along the Mesoamerican Barrier Reef. All capes modeled in this study produced eddy-shedding regimes, but specific eddy attributes differed between spawning and non-spawning sites. Eddies at spawning sites were significantly stronger than those at non-spawning sites, and upwelling and fronts were the products of the eddy formation process. Frontal zones, present particularly at the edges of eddies near the shelf, may serve to retain larvae and nutrients. Spawning site eddies were also more predictable in terms of diameter and longevity. Passive particles released at spawning and control sites were dispersed from the release site at similar rates, but particles from spawning sites were more highly aggregated in their distributions than those from control sites, and remained closer to shore at all times.

**Conclusions/Significance:**

Our findings contradict previous hypotheses that cape spawning leads to high egg dispersion due to offshore transport, and that they are attractive for spawning due to high, variable currents. Rather, we show that current regimes at spawning sites are more predictable, concentrate the eggs, and keep larvae closer to shore. These attributes would confer evolutionary advantages by maintaining relatively similar recruitment patterns year after year.

## Introduction

Fishes use an extraordinarily wide range of strategies for reproduction, each possessing its own delicate balance of tradeoffs between paternal energy expenditure and larval survival. One method of reproduction, often seen in reef fish species, is the formation of large spawning aggregations of up to thousands of individuals [1]. These fish spawning aggregations (FSAs) occur at specific locations and times, and their predictability makes them particularly prone to overfishing as they are easily targeted [2]. For example, aggregations of the commercially fished Nassau grouper *Epinephelus striatus* have undergone severe declines, and reductions in numbers of individuals at aggregations and even extirpations have been documented in Belize [3], Cayman Islands [4], and Mexico [5]. Understanding the biophysical processes taking place at these spawning sites, as well as the negative impacts of FSA loss on these fish stocks, is crucial for the conservation of these species.

Domier and Colin [1] identified two categories of FSAs: *resident*, which assemble at specific times for extended periods throughout the year at locations not far from their home range, and transient, which migrate large distances to aggregate for only a few specific periods of time. Transient spawners expend significant amounts of energy in migration and egg production, and this implies that there exists some significant evolutionary advantage related to the use of the spawning site. Reef fish species in the Caribbean, particularly some groupers (genera *Epinephelus* and *Mycteroperca*) and snappers (genus *Lutjanus*), tend to spawn near certain geomorphological features such as reef promontories, or convex reef outcrops that slope sharply from reefs into deeper water. FSAs have been documented to form at promontories in Belize [6]–[8], the Cayman Islands [9], and Cuba [10]. Promontories technically refer to peninsula-shaped land masses that may or may not be surrounded by a water body, but the structures referred to in the latter examples are nearly entirely submerged (sometimes with the exception of reef crests). Additionally, in the physical sciences the word “cape” is used to describe such features [11]–[12]. To help avoid confusion, we use term “submerged cape” from here on to describe these distinct features, and avoid the misleading term “promontory.”

Much speculation has been put forth in regards to why spawning occurs along these submerged capes and other offshore areas, but few testable hypotheses have been offered. Johannes [13] hypothesized that predation risk was the most influential factor that caused spawning to occur in areas where eggs would be swept offshore from high predation areas. Barlow [14] argued that dispersal of gametes was the adaptive significance of offshore spawning, while Doherty et al. [15] modified this hypothesis and explained how wide dispersal can be advantageous in a patchy marine environment where only a percent of the larvae will come upon suitable food resources. These views have been condensed to form the “off-reef transport hypothesis” where the evolutionary advantage is thought to be in transporting eggs rapidly off reefs, reducing predation, facilitating dispersal, and placing larvae into suitable current regimes [16]. More recently, a growing body of literature has suggested that fish larvae recruit more closely to their source populations than previously thought. Studies based on genetic analyses [17], tagging [18], larval sampling [19], modeling [20], and natal homing behavior [21] suggest that certain mechanisms allow larvae to recruit in environments close to their source populations. However, these studies were based on resident spawners, rather than transient spawners, and species specific studies pertaining to the latter group are lacking.

The specific mechanisms behind cape spawning, and the evolutionary advantages associated with such FSA sites, remain unknown and debated. It has been suggested that these geomorphological features act as cues for “meeting” locations [22], or that fish are attracted to highly variable currents around the edges of these formations [8]. Other authors have noted that larvae tend to be retained near such features, and suggest that mesoscale processes may be at work [23]–[25]. However, hypotheses regarding the significance of the locations of reef fish aggregations near capes have remained untested to date. In this study, we approach this problem by modeling differences in current regimes around capes where spawning is known to occur, and capes with nearly identical geomorphologies where spawning has not been documented to occur. A hydrodynamic model is used to simulate the interaction of dominant current flow with a submerged cape, and separate simulations are run for each site. We also use Lagrangian particle tracking to study the effects of these current flow regimes on the dispersal patterns of passive particles. Attributes not shared by spawning and non-spawning capes (hereafter referred to as “control” sites) can then indicate evolutionary advantages of spawning sites.

## Methods

### Site selection

**Figure 1:**
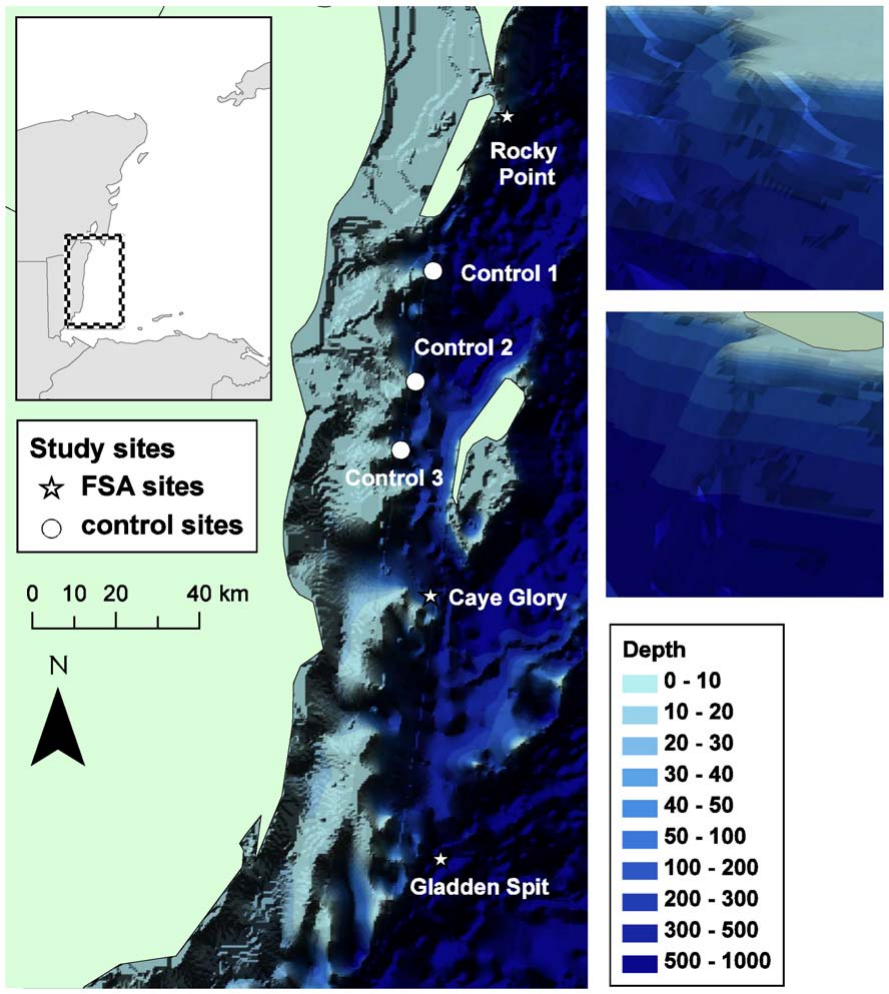
Left: Bathymetry in the Mesoamerican Barrier Reef region with study sites indicated. Upper right: View of Control #2 site from N to S. Lower right: View of Rocky Point site from N to S.

The study area is the Mesoamerican Barrier Reef in Belize, where spawning sites have been relatively well surveyed [6], [8]. Known spawning aggregation sites [6], [8] were overlaid on World Resources Institute (WRI) 500-m resolution bathymetry (integrated at WRI from Coral Reef Millennium Mapping and UNEP-WCMC). Bathymetry was plotted in ArcGIS [ESRI, Redlands CA] and converted into 3 dimensions using the Triangular Irregular Network function, which uses the Delauney triangulation algorithm. Examination of the 3D bathymetry in ArcScene revealed that three known spawning locations along the main barrier reef were located on sinusoidal submerged capes of comparable dimensions (Fig. 1). The existence of these sites as submerged capes has also been confirmed by other studies using alternate sources of bathymetry data [8]. An additional three capes of similar dimensions but where spawning does not occur were identified, and these were chosen as control sites for the study (Fig. 1).

### Leeward eddy dynamics

**Figure 2:**
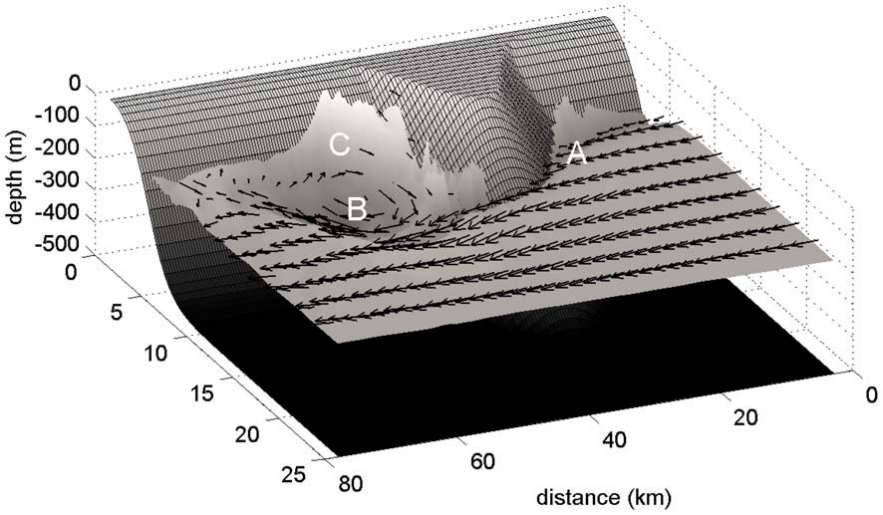
Schematic plot of anticyclone formed by interactions of alongshore current with a submerged cape. Relative vorticity is plotted on cape bathymetry.

To ensure that spawning sites and control sites were comparable in terms of expected current regime, we first calculated the equivalent Reynolds number *Re_f_* associated with each cape, as this has been shown to be indicator of current regime on the leeward side of capes [26]. *Re_f_* is analogous to the Reynolds number, a non-dimensional value which quantifies the relative importance of advective forces to frictional drag forces. Specifically, the *Re_f_* quantifies the relative importance of lateral advection to bottom friction for a flow around a cape [12], [26]. *Re_f_* is given by
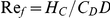
where *H_C_* is the depth of the submerged cape, D is its across-shore diameter, and C_D_ is the bottom drag coefficient (a constant value of 3*10^−3^). At low *Re_f_* frictional forces are dominant, and laminar flows are observed where the flow does not separate from the bathymetry. As *Re_f_* increases, advective forces become relatively more dominant and the flow begins to separate at the tip of the cape, forming a single eddy approximating the width of the cape (Fig. 2). At higher *Re_f_*, eddy shedding regimes may be present, whereby the eddy formed near the tip of the submerged cape sheds off and propagates downstream, and is replaced by another eddy [12].

The presence of a coastline adds friction, and therefore *Re_f_* values for the case of a submerged cape are higher than typical Reynolds numbers for the simple case of flow past a vertical cylinder [26]. In addition to frictional forces, observed current regimes will also be affected by incoming velocity [27], stratification [28], and Rossby number [29]. In this idealized study, we keep the incoming velocity and density profiles constant, focusing only on regime changes due to cape dimensions and slope. However, the Rossby number (*U/(fD*), where *f* is the Coriolis parameter) and Burger number (*R_d_/D)^2^*, where R_d_ is the baroclinic deformation radius) are both dependent on cape diameter *D*, and therefore vary even when incoming velocity is kept constant. The Rossby number, *Ro*, is a dimensionless number indicating the relative importance of inertial forces to rotational forces. When *Ro* >>1, inertial forces dominate cyclogenesis and planetary motions can be neglected, but for *Ro* <1 the effects of rotation are important. Rotation inhibits eddy shedding, and therefore shedding should decrease with decreases in *Ro* [30]. The Burger number, *Bu*, quantifies the importance of stratification in the process of current separation in a rotating fluid [28]. Increasing stratification suppresses vertical motion, so that eddy shedding can occur at smaller values of *Re_f_* when *Bu* is high [30].

### Model set up

We implemented the Regional Oceanic Modeling System (ROMS – UCLA version), [31] to the study of the hydrodynamics at FSA sites. ROMS is a primitive equation, hydrostatic, free surface regional ocean circulation model [31]. It uses a sigma (terrain following) vertical coordinate, which provides higher vertical resolution in shallow areas. ROMS is used to simulate the flow around submerged capes based on actual cape dimensions and incoming modeled flow. Dimensions of the capes were measured in ArcGIS by taking length measurements of the alongshore and across-shore extents at both the 20 m isobath and the seafloor. Vertical slopes at the cape tip and at the adjacent wall were also calculated. These attributes were replicated in the model domain using a sinusoidal cape with increasing amplitude towards depth, and varying widths (frequency of the sine wave) adjusted to fit actual cape dimensions at the 20 m isobath and seafloor. The slope along the tip of the cape and the adjacent shelf were allowed to decrease logarithmically with an average slope equal to the observed bathymetry of the cape and shelf, respectively. Maximum depth of the domain was 500 m or less depending on observed cape depth; all capes had a minimum depth of 20 m.

**Figure 3:**
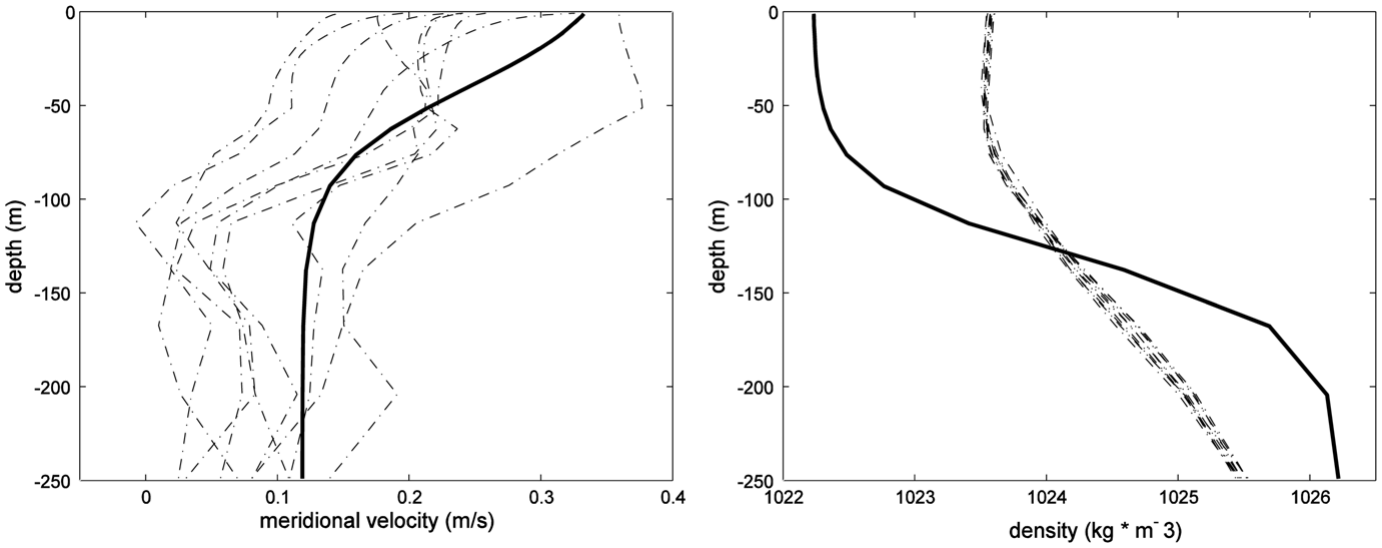
Velocity and density profiles used as initial conditions for the model. Dotted lines: profiles from HYCOM for individual days during the spawning period. Solid line: idealized profile used in the model.

The model domain was rectangular with 430×160 grid cells and 32 depth levels, and a grid cell resolution of 418 m for all sites except for the Rocky Point spawning site and Control #1. These sites had slightly steeper slopes, and resolution therefore had to be increased to 300 m and 362 m respectively to avoid model destabilization in the steeper areas. Vertical resolution at the shelf ranged from 0.2 to 2.4 m, and 1.7 to 51.5 m at maximum depth. We used the Hybrid Coordinate Ocean Model (HYCOM) 2006 hindcast [http://www.hycom.org] to estimate incoming velocity and density profiles. During the winter months, the Belizean section of the Mesoamerican Barrier Reef is subject to large-scale cyclonic circulation leading to a mean annual southward current [32]–[33]. We chose a location just outside the barrier reef at the midpoint of all study sites, and extracted daily velocity and density data from HYCOM for each individual day during the periods of 2 to 7 days after the full moon in the months of January, February and March. This period of time was chosen because multiple species of groupers (*E.striatus, Mycteroperca bonaci, M. venenosa, and M. tigris*) have been observed to spawn at all three FSA sites during these specific periods [6], [34]. We fit the HYCOM velocity and density data to idealized profiles using the methods of Dong et al. [30] in order to initialize ROMS. Profiles were then averaged over all individual days to provide a single density and velocity profile that was used as input for all simulations (Fig. 3). The incoming flow was meridional (parallel to the coast and perpendicular to the cape), set at the northern boundary, and zonal velocity was set to zero.

### Measurements of eddies

Models were allowed to run for at least 50 days at each site. We examined model outputs, excluding the first 5 days of model activity as a “spin up” period, and noted the presence of all eddies with a duration of >3 days (eddies with durations of <3 days tended to be small, turbulent transient features). Because current was from the north and intersected the cape on its anticyclonic side, stable eddies were anticyclonic. Cyclonic eddies were excluded from the analysis as they were only present as smaller unstable features.

In order to characterize size and strength of eddies, we calculated the potential vorticity anomaly, which is a measure of angular momentum within the stratified ocean. In the absence of dissipative effects, for each isopycnal layer, potential vorticity is conserved for each particle of the flow [35]–[36] and can be written as
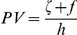



Where *ζ  = ∂_x_v − ∂_y_u* is the relative vorticity, *h* is the thickness of an isopycnal layer, and *f* is the Coriolis frequency. It is also useful to define another quantity which we will refer to as the equivalent quasigeostrophic potential vorticity anomaly (PVA) [37]

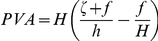
where *H* is constant and represents the unperturbed layer thickness away from the submerged cape. By removing the background vorticity, the potential vorticity gradients are more clearly seen, therefore facilitating the eddy identification and other features produced during their formation such as fronts and filaments.

For each individual anticyclonic eddy, we measured: 1) eddy diameter (width of eddy in the across shore direction, edges defined maximum PVA), 2) eddy longevity (number of days from formation to dissipation), 3) linear velocity (alongshore linear distance traveled by eddy center from formation to dissipation), 4) PVA gradient across the eddy, and 5) number of days between eddy formation. We then statistically compared eddies formed at control sites with those formed at spawning sites in reference to these attributes.

Firstly, we investigated predictability of eddy size, longevity, velocity, strength, and frequency based on the coefficient of variation (CV) of the various eddy attributes. Because variance decreases with increasing sample size, but the number of eddies observed at each site ranged from 5 to 7, we used a subsampling method to calculate CVs for each site. We randomly subsampled 3 eddies from each site and calculated the CV of the subsample for each eddy attribute. This process was repeated 500 times and CVs were averaged across 500 subsamples to form a single measure of within-site variability that was not influenced by sample size. CVs were then compared using t-tests. Secondly, we carried out two-way analysis of variance (ANOVA) to compare the eddy attributes, using individual site and spawning vs. control as factors. Finally, we explored in further detail the mechanics of individual eddies to highlight submesoscale processes that may be important to fish larvae.

### Analyses of Lagrangian transport

Neutrally buoyant particles were released from estimated spawning sites. Spawning of Epinepheline groupers in Belize have been documented to occur at the tip of submerged capes at approximately the 30 m isobaths, in 20 to 30 m of water [38]. Particles were released at the location defined by the intersection of the tip of the cape and the 30 m isobaths, and were released in 20, 25, and 30 m of water during periods of eddy formation. Their trajectories were calculated using the online Lagrangian transport module of ROMS. The code uses a fourth-order accurate Adams-Bashford-Moulton predictor-corrector scheme to integrate dx/dt = u(x,t) (x is the particle coordinate and u its velocity vector) over time given the initial release location and the three-dimensional ROMS velocity fields. The right-hand side is estimated through linear interpolation in time and space of the discrete Eulerian fields, and a random displacement was added to resolve sub-grid scale diffusivity based on Okubo's diffusion diagram [39] using a 300 m diffusion scale equivalent to a dispersion coefficient of 0.05 m^2^ s^−1^. We ran sensitivity analyses and determined that patterns in particle dispersion were only minimally influenced by the depth of release, but were highly influenced by the timing of release due to the state of formation of eddies, which introduces variability in the flow. Due to constraints on computing power, we therefore limited our analyses to releases at a single depth (30 m) at multiple points in time. For each simulation, 200 hundred particles were released daily during all periods of eddy activity. The eddy activity is defined as 2 days before full eddy formation to 1 day after maximum eddy formation. Particle releases were limited to periods of eddy activity since there is evidence of such timing in FSAs [40].

For each spawning site and each daily release, we analyzed the daily locations of the particles for the period of 1 to 10 days post-release. For each day, we calculated: 1) an index for the patchiness of particle distributions, 2) the average distance of particles from the release site, 3) the percentage of particles retained within a 20 km radius of the release site, and 4) the average distance of particles from shore (defined as the location of the particle on the y-axis). To measure particle patchiness, we used the Index of Aggregation, a measure which is not sensitive to relative particle densities and is robust against zero counts in the domain [41]. This index is defined as
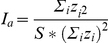
where *z* is the density of particles in each 2-km grid cell *i* , and *S* is the sample area [41]. The grid cell size of 2-km was arbitrarily chosen for the purpose of reporting results, as it was determined that results were not influenced by the size of the grid cell. A lower value of *I_a_* indicates a greater degree of patchiness (i.e., particles are less aggregated). Again, we compared attributes of spawning versus control sites using two-way analysis of variance (ANOVA) using individual site and spawning vs. control as factors.

## Results

Various dimensions of the capes were compared to ensure that control and spawning sites were comparable in terms of expected behavior of the model. The average across-shore extents of the capes ranged from 3 km to 8 km (Table 1). Spawning capes and control capes had average extents of 6 km and 4 km respectively, but the differences in these means were not significant (p = 0.33). The equivalent Reynolds number, *Re_f_*, was similar for all 6 spawning and control capes, with values ranging from 20 to 56 (Table 1). Average *Re_f_* for spawning capes was 33.4 and 33.3 for control capes, and Bu ranged from 4.6 to 32.6 (Table 1). Based on Magaldi et al. [26], capes with such *Re_f_* and *Bu* can be expected to produce moderate to strong eddy shedding regimes. Rossby numbers of <1 (ranging from 0.3 to 0.8) indicate that Coriolis cannot be neglected in this system.

**Table 1:** Dimensions and calculated parameters for study sites.

*Site*	*max depth (m)*	*mean width (m)*	*Re_f_*	*Ro*	*R_d_ (km)*	*Bu*
Rocky Point FSA	500	3,000	55.6	0.8	17.1	32.6
Caye Glory FSA	500	7,000	23.8	0.4	17.1	6.0
Gladden Spit FSA	500	8,000	20.8	0.3	17.1	4.6
Control 1	500	3,750	44.4	0.7	17.1	20.9
Control 2	450	4,500	33.3	0.6	17.1	14.5
Control 3	250	3,750	22.2	0.7	17.1	20.9

FSA  =  fish spawning aggregation; controls are sites where spawning aggregations do not occur. Mean width is cape dimension perpendicular to the coastline. Re_f_ =  equivalent Reynolds number, Ro  =  Rossby number, R_d_  =  baroclinic radius of deformation, Bu  =  Burger number.

**Figure 4:**
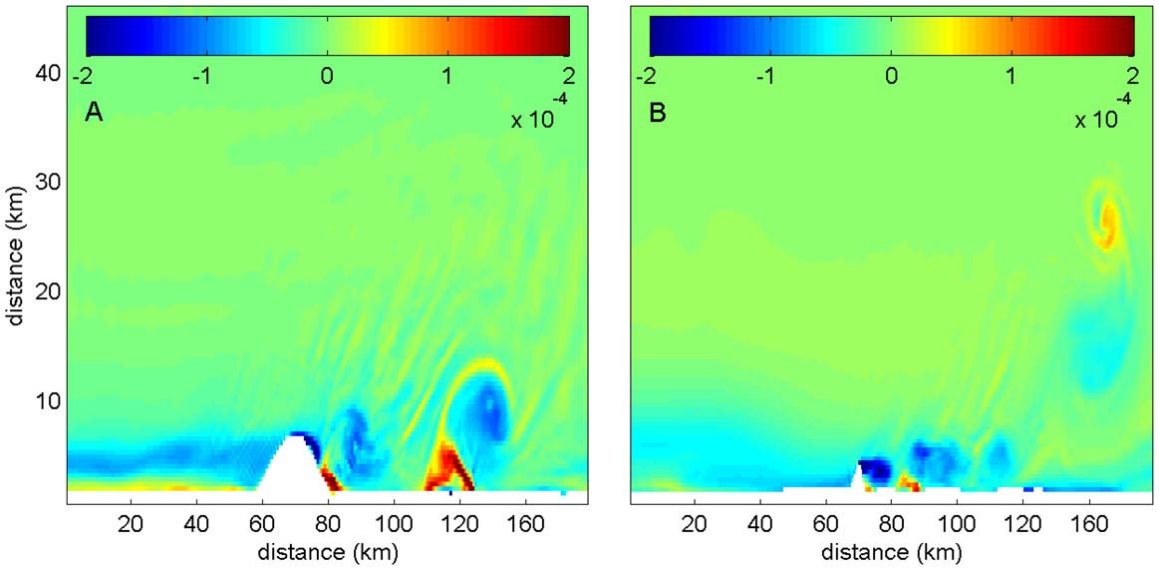
Snapshots of potential vorticity (in m^−1^ s^−1^) of surface layer for (A) Gladden Spit and (B) Control #3.

We tested differences in the CVs of eddy attributes, as low variability in eddy formation should be advantageous for spawning sites to reduce variability in larval recruitment success. The CV for all eddy attributes was lower for spawning capes, and t-tests showed significant differences between the variation in diameter (p = 0.03) and longevity (p = 0.04) of spawning eddies. In other words, eddies spun up at spawning sites were more predictable in terms of their size and duration, while eddies at control sites were more variable in these characteristics (Fig. 4). Differences in CVs were not significant for linear velocity (p = 0.20), maximum PVA gradient (p = 0.36), or frequency of eddy formation (p = 0.22). Due to the nature of our study our sample sizes were low (N = 3), and the lack of significance in the differences in predictability in these latter attributes is not necessarily meaningful. We also tested differences in eddy attributes across sites and for spawning versus control sites using a two-way ANOVA. Eddies at spawning sites had significantly higher PVA gradients than those at control sites (p = 0.002). No significant differences were found between spawning and control sites for other eddy attributes (diameter, longevity, linear velocity, frequency; Table 2).

**Table 2:** Results of 2-way analysis of variance for differences in eddy attributes between sites and for control vs. spawning sites.

*eddy attribute*	*mean value FSA sites*	*mean value control sites*	*site effect* *Pr(>F)*	*control vs. spawning site* *Pr(>F)*
diameter (km)	18.5	15.3	0.45	0.20
longevity (days)	5.9	6.3	0.46	0.61
linear velocity (km/d)	13.3	13.2	0.55	0.95
Δ potential vorticity (m^−1^ s^−1^)	5e-04	3e-04	*0.03	**0.002
frequency (d. between formation)	4.2	5.4	0.50	0.46

**Figure 5:**
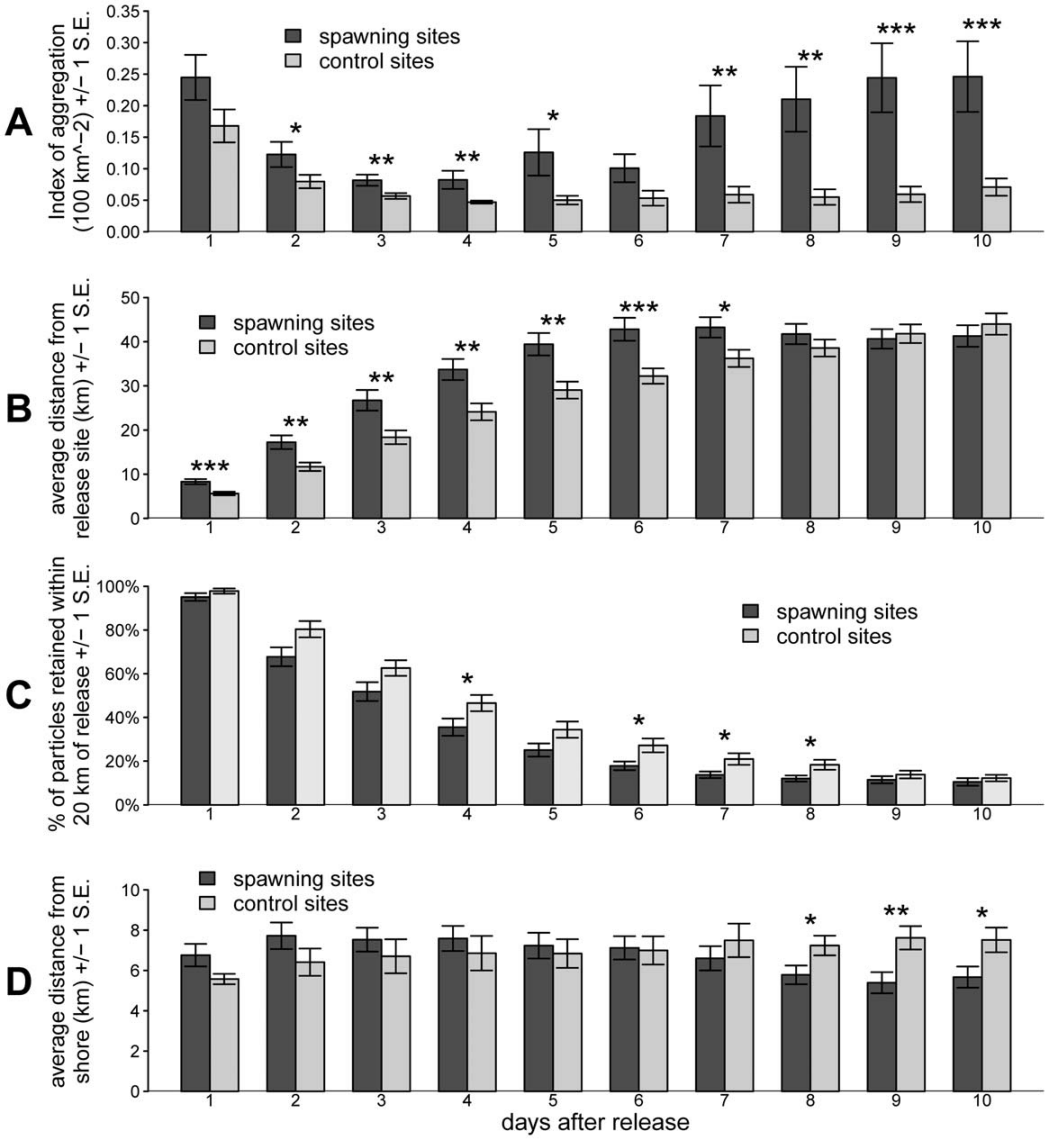
Differences in attributes of passive particles released at spawning versus control sites for 1 – 10 days post release. A) index of particle aggregation. B) average distance of particles from release site (km). C) percentage of particles retained within 20 km of release site. D) average distance of particles from shore (km). *p<0.05, **p<0.01, ***p<0.001.

**Figure 6:**
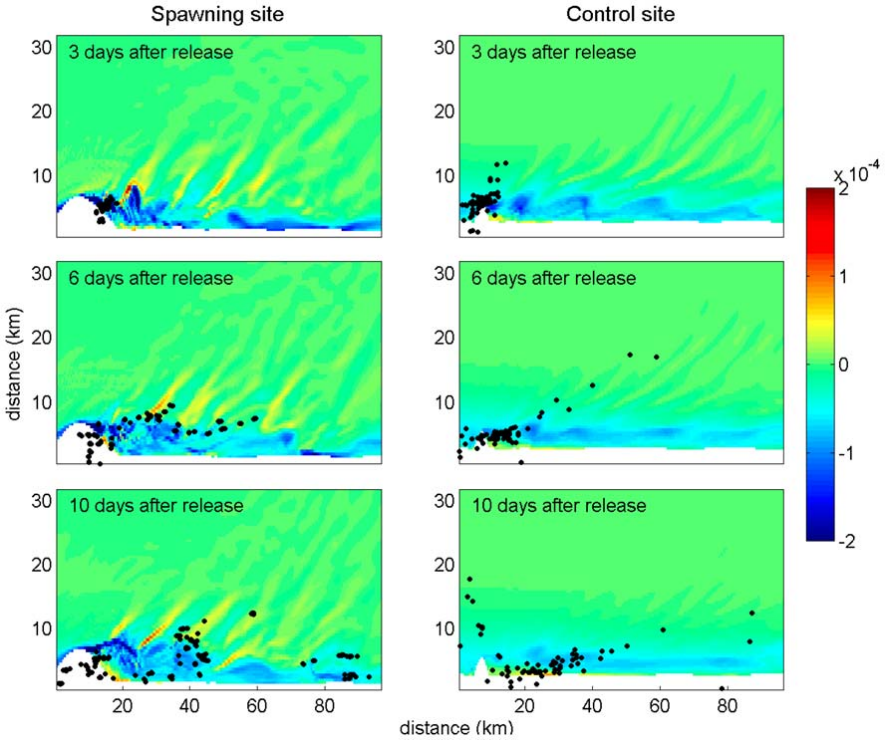
Series of snapshots showing eddy development and transport of passive particles. Locations of particles 3 days, 6 days, and 10 days after release are shown for a spawning site (left) and control site (right). Particle locations are plotted overlaying potential vorticity field (m^−1^ s^−1^) in the surface layer.

We tested differences in the dispersion patterns of passive particles released from spawning sites in regards to patchiness, dispersion, and distance from shore. Particles released from spawning sites were significantly more aggregated than particles at control sites during the entire study period, with the exception of days 1 and 6 post-release (Fig. 5a). After day 6, differences became increasingly apparent; by day 10 post-release, differences in patchiness were maximized. At this time, the index of aggregation at control sites was 0.07+/− 0.01 km^−2^, while the index of aggregation at spawning sites was 0.25+/− 0.06 km^−2^, indicating much more aggregated distributions at spawning sites (p<0.001). Particles at both control and spawning sites tended to be concentrated in areas of high potential vorticity (Fig. 6).

Particles were initially dispersed on average farther from the spawning site than those from control sites; however, these differences disappeared after 7 days post-release (Fig. 5b, c). At one day post-release, particles released at spawning sites were dispersed significantly further from the release site than particles at control sites (p<0.001). These differences increased and were maximized at day 6, when particles at spawning sites had dispersed an average of 42.8 km from the release site, while particles at control sites had dispersed an average of 32.2 km. These differences decreased at day 7 and were non-significant at day 8. By day 9, dispersal at control sites was greater than spawning sites, though these differences were not significant (p = 0.170). From days 4 to 8 post-release, the percentage of particles retained within 20 km from the release location was significantly greater at control sites (p<0.05), but these differences disappeared by day 9 post-release (p = 0.36, Fig. 5c).

There were no significant differences in nearshore retention between control and spawning sites for the first 7 days post-release (Fig. 5d). By day 8, however, particles released at spawning sites were retained significantly closer to shore (p = 0.03). This pattern continued through days 9 and 10. At day 10, particles released at control sites were at 7.5+/− 0.6 km from the shore, while those released at spawning sites were at 5.7+/− 0.5 km from shore.

## Discussion

Our major finding is that eddies formed at spawning capes consistently favored processes of nearshore retention and concentration in comparison to eddies formed at similar capes where spawning does not occur. Eddies formed at spawning capes were significantly less variable with respect to diameter and longevity as compared to non spawning sites. Predictability of currents should be a desirable attribute for spawning sites, as it would be advantageous for individuals to release eggs in a location where survival of some larvae would be ensured. We also found that eddies formed at spawning capes have significantly greater potential vorticity anomaly (PVA) gradients. These strong PVA gradients are associated with increased rates of upwelling and accumulation of passive particles. Furthermore, these eddy attributes had demonstrable effects on passive particles released upstream. Particles released at spawning capes were significantly more clumped and were retained closer to shore in comparison to particles released at control sites.

We first discuss the mechanics of eddies formed in our study, and detail how and where submesoscale nutrient upwelling occurs. We then describe the specific aspects of eddies that pertain to the survival and retention of passive particles such as eggs and nutrients. Finally, we conclude with a discussion of how our findings are important to management of spawning aggregation sites.

Leeward eddy formation is a result of alongshore current separation which occurs in the presence of obstacles [12]. When an alongshore current encounters a submerged cape, the water mass is forced to pass through a smaller area, and flow accelerates as it reaches the tip of the cape. On the leeward side of the cape, a pressure gradient is set up, with high pressure produced at the upstream face of the cape (Fig. 2, location A) and lower pressure in the shadow of the cape (Fig. 2, location B). Due to this pressure gradient, eddies can be formed, in cases where the inertial force of the incoming current is greater than the frictional drag between current and topography. For our case, where a southward current is intersecting a cape located on a western shelf, stable anticyclones will occur. Cyclonic eddies can also occur as a result of shear along the edges of anticyclones [26].

Submesoscale nutrient upwelling occurs as a result of 1) lifting isopycnals and 2) frontogenesis where density gradients are sharp [42]. In the northern hemisphere, Coriolis force points to the right of a geostrophic current, or towards the center of an anticyclone. Coriolis force in an anticyclone is balanced by centrifugal and pressure forces pointing outwards from the anticyclone. The balance of these forces creates a sea surface high in the surface layers of an anticyclone, and a deepening of isopycnals in the subsurface layers (see Fig. 2, center of anticyclone at B). As the eddy develops, surface isopycnals are uplifted and subsurface isopycnals deepen, and as the eddy deteriorates, isopycnals flatten. Through this mechanism, nutrients can be brought up to the surface layers. Frontogenesis, another process important for upwelling, occurs where the PVA gradient is large as a result of sharp density gradients. Submesoscale upwelling occurs in these regions as a result of strong surface shear [42]. Filaments of high positive vorticity originate near the coast where the PVA gradient is maximized (Fig. 2, between B and C), and are entrained in the eddy on its upstream edge (Fig. 4a).

### Significance of eddy mechanics to larvae: Ocean triads

Bakun [43] observed that spawning habitats of different species shared many of the same attributes, and proposed the ‘ocean triad’ concept to describe suitability of spawning habitat. This theory has been used to describe habitat for a number of temperate species including tunas [43], anchovies [44], and other small pelagic species [45]. Bakun proposes that three processes make up suitable habitat: 1) nutrient enrichment, 2) concentration of nutrients and larvae, and 3) retention of larvae near suitable habitat. We discuss our findings in light of this theory and address each of the mechanisms individually.

### Enrichment

**Figure 7:**
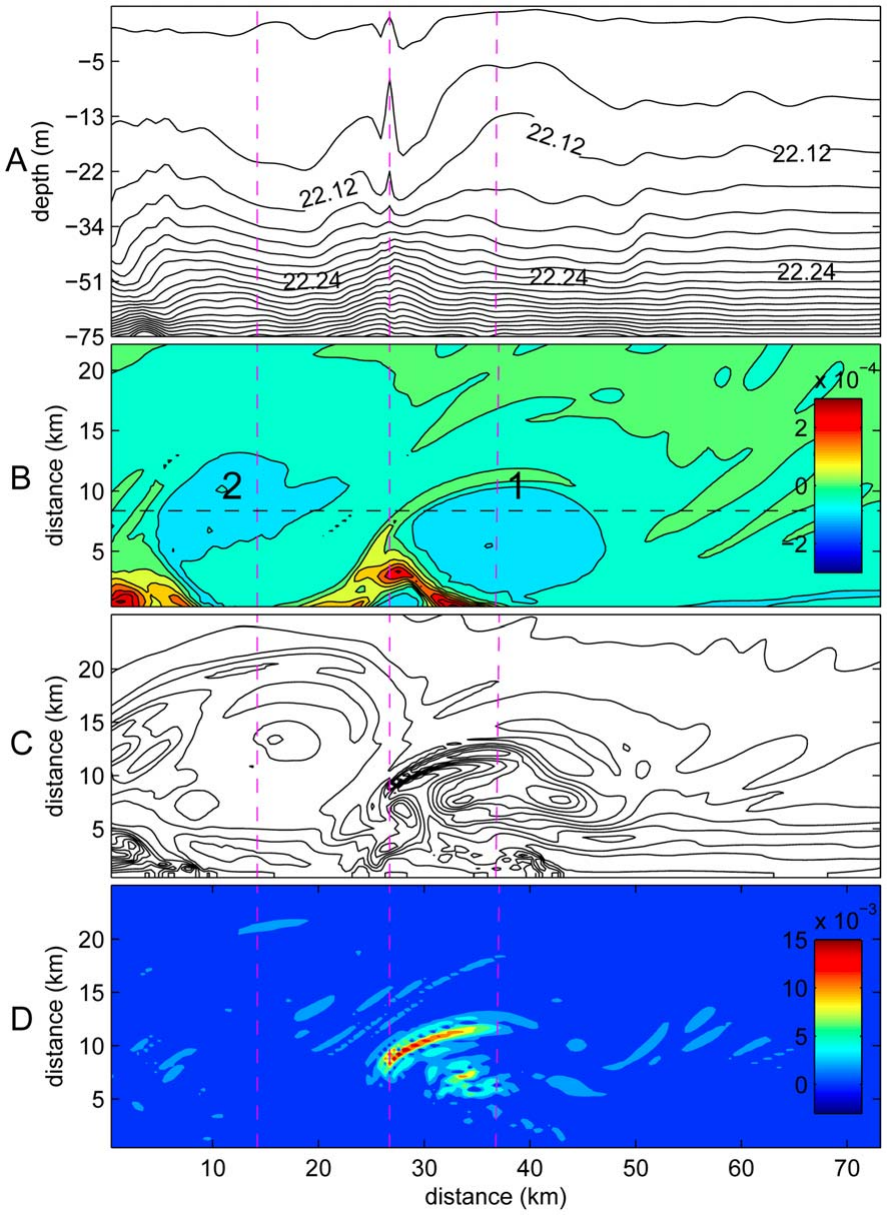
Snapshot of Gladden Spit model output showing important physical features and processes of eddy formation. Pink dotted lines are added for ease of visualizing behavior at eddy edge vs. eddy centers. Cape (not shown) is to the left of the extent. A) Isopycnals (density anomalies in km m^−3^) plotted in an alongshore cross section at y = 20 (black dashed line in B). B) Plot of potential vorticity values (in m^−1^ s^−1^) in the surface layer to view the presence of eddies. Red indicates positive vorticity; blue indicates negative vorticity. C) Contours of isopycnals; concentrated lines denote frontal zones. D) Plot of absolute vertical velocities (m s^−1^) * density anomalies (kg m^−3^) in the surface layer. Red indicates vertical movements.

Eddies convert mechanical energy to biological energy [43] and can restructure small scale habitat in offshore areas. For the case of the eddies formed at the spawning sites in our study, enrichment appears to occur primarily as a result of 1) uplifting isopycnals and 2) frontogenesis, as discussed above. Vertical movement associated with the passing of two eddies can be seen in a cross section of isopycnals (Fig. 7a) and also in a plot of w*rho, indicating vertical velocities (Fig. 7d). In these figures, Eddy 2 is still gaining strength and not yet spun up completely, and vertical movement is at a minimum. Eddy 1 has reached its maximum strength and vertical movement can clearly be seen in the center of the anticyclone, where water masses from depths of approximately 25 to 30 m can be pushed up to depths of about 10 m (Fig. 7a). This phenomenon has been observed in other modeling studies; Lévy [42] found that new production is higher in anticyclones than cyclones due to upwelling within anticyclone cores.

Frontogenesis due to sharp gradients in densities occurs most frequently on the upstream side of anticyclones (e.g., Fig. 7 at 27 km). Positive potential vorticity filaments tend to be longer and better defined in eddies at spawning capes (Fig. 4). These results suggest that enrichment by injection of nutrients into the surface layers is a characteristic of suitable larval habitat. How exactly this nutrient input may create an advantageous environment for larval survival remains unknown, due to the inherent difficulties in directly sampling the spatial characteristics of submesoscale eddies. In a modeling study, Lévy [42] showed that new production was enhanced in areas of strong vorticity gradients, and that patterns of phytoplankton abundance resembled patterns in new production. Submesoscale variation in plankton abundance is produced by ageostrophic upwelling due to horizontal transport [42]. This enrichment process differs from plankton blooms that may occur due to vertical mixing within mesoscale eddies [46]–[47]. Regardless of the specific mechanisms producing patterns in plankton abundance, there is some limited evidence that fishes may exploit these patterns. Richardson et al. [48] showed that spawning of sailfish (*Istiophorus platypterus*) appeared to occur in a location and time that resulted in larvae occupying areas of favorable feeding habitat within an eddy. The results obtained in our study suggest that the Nassau grouper may also potentially be spawning in specific locations so that suitability of larval feeding habitat is maximized.

### Concentration

Concentration of both nutrients and larvae can occur from both vertical and horizontal movements within eddies. Potential vorticity acts as a tracer; i.e., passive particles would be expected to become concentrated in areas of positive potential vorticity. In our simulations, passive particles such as nutrients and eggs indeed tend to be concentrated within positive vorticity filaments formed along the edges of anticyclonic eddies (Fig. 6). Particularly as the eddy strengthens, bands of upwelling and sinking occur on scales of <1 km (Fig. 7d). A noteworthy feature of the eddies in our study is that their axes are not vertical; rather, they are shifted so that the bottom points downstream. This asymmetry can also be seen in the isopycnals; the top of the eddy is further upstream than at the bottom (Fig. 7a). PVA gradients are also asymmetrical and are stronger on the upstream edge of the eddy. The upstream edges of the eddies in particular appear to concentrate eggs and nutrients and may be of particular importance for the survival of larvae hatching within.

Indeed, our simulations reveal a greater degree of aggregation of passive particles at spawning sites as compared to control sites, particularly in the upstream edges of eddies (Fig. 6). Particles released at spawning sites tend to stay concentrated in smaller areas near positive vorticity filaments, whereas particles released at control sites tend to be dispersed more patchily. Mechanical activity of eddies, which serves to concentrate passive particles such as larvae and prey into smaller areas of the pelagic habitat, can potentially facilitate feeding activity by increasing encounter rates between larvae and food particles. This would be particularly important in pre-flexion larvae with limited swimming abilities. Larvae of Nassau grouper (*E. striatus*) begin feeding after approximately 4 days after egg fertilization, and develop swimming abilities around 13 days [49]. Notably, significant differences in particle patchiness between spawning and control sites appeared at day 2 post-release, and continued through the 10-day post-release period (Fig. 5a). Concentration of larvae in smaller areas, particularly in areas likely to be injected with nutrients through the processes described above, may assist larvae in feeding and contribute to the survival during the delicate pelagic life stage.

### Nearshore Retention

Retention of larvae in leeward cape eddies has been noted [23], [25], but this process is ephemeral. Thus far, no study has reported retention as limited dispersion from shore. Filaments of large positive PVA surrounding eddies are the products of strong coherent eddies, which have a longer lifespan [42]. These filaments were generated at the edges of spawning eddies (Fig. 4a) but occurred less frequently around eddies from the control site eddies (Fig. 4b). Eddies at spawning sites remained attached to the shelf slope without exception, while the eddies at control sites occasionally traveled offshore (Fig. 4b). These eddies have an effect on the dispersion patterns of passive particles; after 10 days, particles released at spawning sites remained significantly closer to shore. Thus, the eddies appear to not only retain larvae at their periphery, but also keep them relatively close to potential settlement habitat. The retention of eddies close to shore may be an important feature that allows larvae to sense and reach settlement habitat once swimming ability is developed and the pelagic phase ends.

The mechanical retention mechanisms of eddies also appear to have a synergistic effect with larval swimming behavior in the early stages. Sensitivity analyses by Paris . [50] showed that eddies and larval vertical migration combined have a significant shrinking effect on the dispersal kernel (probability density function of dispersal distances) in the Mesoamerican Region. Thirty-day-long simulations of passive particles in surface currents without eddies suggested that peak recruitment generally occurs at about 100 km from the larval source. In the presence of coastal eddies, dispersal was reduced by about half this distance. When larval ontogenic vertical migration is simulated within flow fields with eddies, the distance shrunk further to less than 20 km. The concentration mechanisms described above are also likely to be enhanced by larval behavior.

Many adaptive tradeoffs exist in spawning site selection: predation avoidance versus access to food supply, retention close to a known suitable habitat versus dispersal to propagate genotypes across wider geographical regions, constant small supply of gametes throughout an extended period versus a only a few large gamete releases per year, and energy expenditures in migration versus suitability of local habitats. The Nassau grouper, *Epinephelus striatus*, appears to be a relatively extreme example of such tradeoffs, as they sometimes undertake migrations of up to a month to travel over 100 km [51] to sites where they release all of their eggs and sperm in only a few events each year [34]. Nassau grouper spawning sites are often located at capes where anticyclonic eddies would be expected to appear [8], [52]. Such excessive energy expenditures would need to be outweighed by some adaptive mechanism of the spawning site, such as increased larval survival. Notably, *E. striatus* develops elongated dorsal and pelvic spines during the early stages of its pelagic phase [53]. We propose that this species is particularly well adapted for larval development within the high predation environments that would occur in these eddies due to nutrient enrichment and concentration processes.

While we did not investigate to a great extent the differences between individual spawning sites, the Gladden Spit site is worth mentioning in that the vorticity gradients of eddies were significantly higher in this site than the other sites (p = 0.03). It was also the most predictable site in terms of eddy shedding frequency, with stable anticyclones being formed every 2 to 7 days. Gladden Spit is the most widely used multi-species spawning site in Belize, with over 20 species migrating to spawn at different times of the year [6], [54]. Whale sharks, *Rhincodon typus*, are also consistently present in this area, and a large ecotourism industry has developed around their predictable arrival [7]. The wide use of the site by a number of different species may be a result of the predictability of favorable current regimes, as well as upwelling created by uplifting isopycnals at the surface layers. This would serve to supply nutrients to the planktonic food chain.

### Management implications

Fish spawning aggregations are in need of protection, as overfishing these aggregations can promote not only ecological damage, but also damage to livelihoods of reef dependent people [2]. Understanding the biophysical processes influencing spawning is important to inform management actions that need to take place for conservation of commercial fish stocks. It has been proposed that cape spawning sites are advantageous because they will promote stochastic offshore transport [8], [22], but our results contradict these findings. The distinction is important to make as there are implications for management of fish stocks and spawning aggregations. If these spawning sites are indeed selected because of an evolutionary advantage in larval survival rates, as our results suggest, there may be a synergistic negative effect to the fish population associated with higher exploitation rates at the most productive sites. Stocks are impacted by not only the loss of spawners when these sites are exploited, but overall larval survivorship rate can be lowered if these “prime spawning locations” are fished out while “suboptimal spawning locations” may persist. It is unclear whether fish have the ability to reform spawning aggregations after they have been extirpated at a location. Given the predictability of eddies shed at spawning capes and the apparent evolutionary advantage associated with this predictability, we have reason to believe that extirpated sites could potentially repopulate after a period of protection. In the absence of more information, fishery managers may be advised to protect these sites, even after their extirpation.

Our findings contradict previous hypotheses that cape spawning leads to high egg dispersion due to offshore transport, and that they are attractive for spawning due to high, variable currents. Rather, we show that current regimes at spawning sites are more predictable, which would confer evolutionary advantages by maintaining relatively similar recruitment patterns year after year. PVA gradient differences (i.e., eddy strengths) are also higher in spawning eddies, suggesting that the influx of nutrients at a site is an advantageous enrichment attribute. Fronts also serve to retain and concentrate larvae in areas of expected nutrient input, potentially facilitating larval feeding and survival in the earlier critical stages. Finally, spawning eddies retain larvae nearer to shore, assisting in recruitment to suitable habitats once swimming ability is developed. By providing a mechanistic understanding of current regimes around spawning capes, our study suggests that important processes are occurring at the submesoscale. If submesoscale processes important to marine larvae are not adequately resolved, this may result in large inaccuracies in estimating marine population size and connectivity [55]. This is especially critical for large predator fishes that have been depleted worldwide, causing top-down changes in the marine ecosystem. With the recent growth in the use of spatial management tools in the marine environment, it is crucial to have a better understanding of processes relating to larval survival and dispersion.
